# Swine Norovirus: Past, Present, and Future

**DOI:** 10.3390/v14030537

**Published:** 2022-03-05

**Authors:** Lara Cavicchio, Andrea Laconi, Alessandra Piccirillo, Maria Serena Beato

**Affiliations:** 1 Diagnostic Virology Laboratory, Istituto Zooprofilattico Sperimentale delle Venezie (IZSVE), Viale dell’Università 10, Legnaro, 35020 Padua, Italy; lcavicchio@izsvenezie.it; 2 Department of Comparative Biomedicine and Food Science, University of Padua, Viale dell’Università 16, Legnaro, 35020 Padua, Italy; andrea.laconi@unipd.it (A.L.); alessandra.piccirillo@unipd.it (A.P.); 3 National Reference Laboratory for African Swine Fever and Ruminant retroviruses, Istituto Zooprofilattico Sperimentale dell’Umbria e delle Marche (IZSUM), Via G. Salvemini, 1, 06126 Perugia, Italy

**Keywords:** norovirus, swine, detection, zoonosis

## Abstract

Norovirus, an ssRNA + virus of the family *Caliciviridae*, is a leading disease burden in humans worldwide, causing an estimated 600 million cases of acute gastroenteritis every year. Since the discovery of norovirus in the faeces of swine in Japan in the 1990s, swine norovirus has been reported in several countries on several continents. The identification of the human-associated GII.4 genotype in swine has raised questions about this animal species as a reservoir of norovirus with zoonotic potential, even if species-specific P-types are usually detected in swine. This review summarises the available data regarding the geographic distribution of norovirus in swine, the years of detection, the genotype characterisation, and the prevalence in specific production groups. Furthermore, we discuss the major bottlenecks for the detection and characterisation of swine noroviruses.

## 1. Introduction

The World Health Organisation (WHO) stated that in the last decade, approximately 75% of new diseases in humans are caused by pathogens from animals or animal products. Studies reported that approximately 60% of the 1400 known human pathogens are zoonotic, and 75% of the 175 species that are responsible for serious disease outbreaks in humans are of zoonotic origin [1]. Globalisation has led to extensive, continuous, and rapid movement of people, as well as of animals and animal products, contributing to the spread of pathogens. Therefore, it is of absolute importance to investigate the role of animal reservoirs in the maintenance and spread of zoonotic pathogens or with zoonotic potential. Emerging Infectious Diseases (EIDs) continue to threaten human populations and their frequency and severity are likely to increase. EIDs are characteristic of a complex ecosystem that involves human populations, their lifestyle and migratory flows, livestock, and wild animals. Furthermore, anthropogenic activities, which cause changes in land use and global climate, are creating new areas of interaction between wild species, domesticated species, and human populations. In this context, the emergence of new EIDs is promoted and facilitated. Furthermore, the ongoing COVID-19 health crisis has highlighted the crucial role of animal reservoirs in the emergence of new pathogens with pandemic potential and, therefore, the importance of studying infectious agents in these reservoirs. As a matter of fact, coronavirus, rotavirus, and norovirus are viruses that circulate in both humans and animals. Worldwide, human norovirus (HuNoV) causes an estimated 685 million cases of gastroenteritis and is the most common cause of acute gastroenteritis since about one in five cases is caused by HuNoV [2]. About 200 million cases are reported in young children (<5 years of age), resulting in 50,000 child deaths every year, mainly in low-income countries [2]. However, HuNoV infection represents a burden in high-income countries with an estimated cost of USD 60 billion worldwide due to healthcare costs and loss of productivity. In 2020, HuNoV and other caliciviruses were the fourth most frequently reported causative agents of food and waterborne outbreaks (FBO) in the EU, associated with 130 outbreaks in thirteen member states (i.e., Belgium, Czechia, Denmark, Finland, France, Germany, Italy, Latvia, Malta, the Netherlands, Poland, Spain, and Sweden). In the EU, HuNoV was associated with large outbreaks (20.4 cases on average), and most of them were classified as general outbreaks (*n* = 82; 63.1%). In 2020, six outbreaks involved more than 100 cases each. Furthermore, two outbreaks identified in Denmark were reported to be part of the same multi-country outbreak related to oyster consumption [3]. In the present review, we summarised the data available on norovirus in swine (swine norovirus); studies investigating merely the presence of *Calicivirus* without further characterisation were not considered.

## 2. Results

### 2.1. Viral Structure and Genome Organisation

Noroviruses are small, non-enveloped viruses of the *Caliciviridae* family. The name *Caliciviridae* derives from the Latin word calix and is due to the characteristic cup-shaped depression on the virion surface of all the family members [4].

The *Calicivirus* virion has a diameter of 27–40 nm, and the capsid is composed of 90 dimers of the single, major capsid protein (VP1), which are arranged in a T = 3 icosahedral symmetry [4]. The VP1 protein has a shell (S) domain, conserved and internal to the virion, and a protruding domain (P), variable and externally exposed. This last domain is further composed of P1 and P2 subdomains. The hypervariable region present in P2, interacting with the receptors on the host cell’s surface, is particularly important for classifying the antigenic diversity of the members of this viral family [4]. The virions also contain a minor basic protein (VP2) associated with the VP1 S domain. VP2 is essential for the production of infectious virions and enhances the stability of VP1.

**Figure 1:**
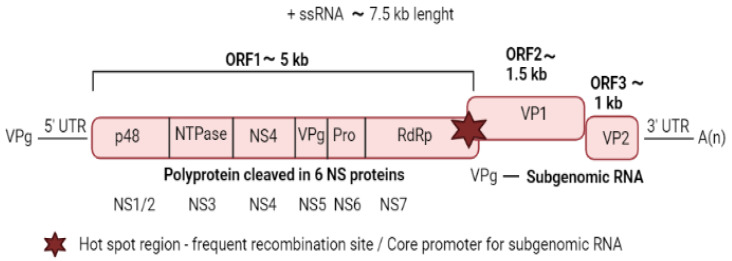
Genome structure of norovirus. The figure represented the genome organisation of norovirus. The polyprotein encoded by ORF1 is post-translationally cleaved by the virus-encoded protease, Pro (also known as NS6 or 3C-like), into individual proteins: p48 (also known as NS1/2 or N-term), NTPase (also known as NS3 or 2C-like), p22 (also known as NS4 or 3A-like), VPg, Pro and RNA-dependent RNA polymerase (RdRp). Subgenomic (+) RNAs contain only ORF2 and ORF3 and are used for the production of VP1 and VP2. Created in Biorender.com (https://biorender.com/, accessed on 2 February 2022).

Noroviruses are characterised by a linear single-stranded positive-sense RNA of about 7.5 kb with a polyadenylated tail at the 3′ end [5] (Figure 1).

Meanwhile, the 5′ end of the viral genome is linked to the viral protein (VPg), together with a subgenomic RNA (sgRNA) corresponding to the last 2.3 kb of the genome [6]. Highly conserved short untranslated regions (UTRs) are present at the extremities of the viral genome and repeated throughout flanking the coding regions [7]. These UTRs play a vital role in viral replication, translation, and pathogenesis and are known to be able to bind to both viral and host factors [8]. The norovirus genome presents three open reading frames (ORFs). The first ORF, from the 5′ end, is the ORF1 encoding a large polyprotein, which is cleaved into six nonstructural proteins (NS). The NS1/2, NS3, and NS4 are involved in the formation of the replication complex, NS5 (or VPg) is linked to the genomic (gRNA) and the sgRNA, NS6 cleaves the polyprotein, and NS7 (or RdRp) are involved in the replication of the viral genome [5]. ORF2 and ORF3 are located near the 3′ end of the genome and encode the VP1 and VP2, respectively, which are both structural components of the virions [5]. The prototypic strain of the norovirus genera was first detected in 1968 in Norwalk city (Ohio) during an acute gastroenteritis outbreak in humans, while in animals, it was detected for the first time in calves in 1978 [9].

### 2.2. Virus Replication

In vitro cultivation of HuNoVs was achieved only recently by using intestinal enteroids [10,11,12]; however, for swine noroviruses, there are no available in vitro cultivation techniques, and therefore most of the information on their biology derives from studies on similar cultivable viruses, such as murine noroviruses or other caliciviruses [13]. Before the advent of intestinal enteroids, for studying the norovirus life cycle, human strains were used to infect gnotobiotic pigs with good results [14,15,16,17]. To the best of our knowledge, similar studies are not available for swine norovirus. However, given its similarity to the HuNoV, a similar replication cycle may be assumed, but further studies should be conducted to confirm this hypothesis.

The attachment of norovirus to the cell surface is mediated by the VP1 and carbohydrate cell receptors; in particular, the histoblood group antigens (HBGAs) seem to have a crucial role [18]. CD300lf, an integral membrane protein containing an immunoglobulin domain, which binds to the P2 subdomain of VP1, mediates the entry into the cell of murine norovirus. However, the analogous receptor for the other noroviruses and the mechanism of endocytosis is still unknown [18].

The virus translation and transcription mechanism occur in the cytoplasm. After uncoating and disassembly, the VPg-linked RNA acts as a messenger RNA (mRNA) for the initial round or translation [4]. VPg (NS5) works as a cap and mediates the translation of viral RNA into proteins using the host cell apparatus. ORF1 encodes a polyprotein that is co- and post-translationally cleaved by a viral protease (NS6) to release viral proteins and their precursors (NS1/2 to NS7) [18]. Subgenomic RNA is expressed at higher levels than genomic RNA; this could be a strategy to increase the production levels of VP1 for the assembly of the virus [18].

Viral replication occurs in close association with the rearrangement of intracellular membranes driven by viral proteins NS1/2 and NS4 [18]. Genome duplication occurs through the synthesis of an intermediate RNA negative sense, which serves as a template for the RNA-dependent RNA-polymerase (RdRp) that synthesises positive sense gRNA and sgRNA [4]. The mechanism of virus release is still unclear.

### 2.3. Studying Swine Norovirus: When and How Frequent

The present section provides information on the number of studies dedicated to investigating the presence of swine norovirus worldwide, highlighting countries for which more reports are available.

The detection of swine norovirus dates back to 1997 when Sugieda et al. [19] reported for the first time the presence of norovirus particles in swine faeces collected in Japan. Since then and until 2022, 47 studies investigating the presence of swine norovirus around the world have been published, including studies that did not detect swine norovirus. The investigations of swine noroviruses were either the result of retrospective studies on archived swine faecal samples [20,21,22,23,24,25,26] or studies with ad hoc sampling strategies to investigate the presence of swine noroviruses in a target geographic area [27,28,29,30,31,32,33,34,35,36,37,38,39,40,41,42,43]. Both types of studies present different sampling approaches, hampering a straightforward data comparison. Indeed, the information available, although increased over the years, is fragmented, and, most importantly, some studies represent single reports without any follow-up investigations (i.e., studies conducted in Belgium [44], Slovenia [34], Spain [31], Hungary [37] and Greece [45], Ethiopia [41], Taiwan [46], South Korea [32], Venezuela [47]). Only a few countries made more effort in detecting and characterising swine noroviruses, and two or more studies are available for each of these countries (i.e., Italy [21,22,28,48], USA [13,20,40,49,50], Canada [26,48], China [39,49,50,51], Japan [19,20,35,36,52,53,54], Germany [33,55], The Netherlands [55,56], Brazil [25,29,30,57]). It appears that efforts to investigate the circulation of swine norovirus should be implemented in all continents.

### 2.4. Where and When: Countries, Years of Detection and Seasonality

In this section, information on the detection of swine norovirus is summarised. Table 1 summarises the countries reporting swine norovirus. Furthermore, at the end of this section, studies reporting serological evidence of swine norovirus are reported.

To date, swine noroviruses were reported in six continents, i.e., Asia, Europe, Africa, North and South America, and Oceania. After the first detection in Japan in 1997, two retrospective studies investigated the presence of swine norovirus in samples collected before 1997 [20,47]. Martinez et al. screened archived samples collected in 1993 in Venezuela [47], while Farkas et al. investigated samples collected in 1997 in the United States [20]; in both studies, swine norovirus was not detected. Since the first identification of swine norovirus, the virus has been detected in China (four studies [38,39,49,50], in North and South Korea (three studies [32,62,63]), Taiwan (one study [46]), and Japan (other four studies). Eighteen studies on swine norovirus in North and South America are available [13,23,25,29,30,40,43,47,48,57,58,59,62,63,64,65,66,67,68], and the first detections in this continent occurred in the United States [43]. In the United States and Canada, the presence of swine norovirus in swine populations was investigated in seven [5,8,27,30,37,38,41] and four studies [26,61,64,69], respectively, although two out of the six studies carried out in the United States did not detect any virus circulation. Swine norovirus was detected in Brazil, Nicaragua, Bolivia, Colombia, and Venezuela [29,30,47,57,67,68]. Notably, swine norovirus was also detected in illegally imported pork meat in the European Union (EU) from Venezuela, Bolivia, and Brazil [68]. The detection of swine norovirus in the African continent is relatively recent since the virus was reported in 2016 in Ethiopia [41]. Swine norovirus was also reported in Oceania (New Zealand) more than ten years ago [60]. Thus far, eight European countries have detected the presence of swine noroviruses in their swine populations over a 21-year period between 1998 and 2019 [21,22,24,28,31,33,34,44,45,48,59,60,70]. Italy has generated six studies on swine noroviruses between 2008 and 2019, reporting the first detection in 2014 while investigating samples collected between 2006 and 2007 [24]. The Netherlands and Germany were the second EU countries to generate multiple studies on swine norovirus [33,59,60]. For each of the other EU countries reporting swine norovirus, i.e., Belgium, Slovenia, Spain, and Hungary, only one study is available [31,34,42,44]. Furthermore, sequence data on a strain of swine norovirus identified in a sample collected in 2009 in Denmark are publicly available. In conclusion, the comparison of swine norovirus prevalence between countries and/or continents may not be straightforward due to different sampling strategies employed (e.g., different seasons, different productive groups, retrospective studies vs. active surveillance). However, the prevalence in Europe and Asia seems comparable (0.5–18.9%, mean = 5.9% and 0.22–18.26%, mean = 4.49%, respectively), while in North America it seems slightly higher (2.1–25%, mean = 9.37%).

Studies assessing the impact of seasons on the prevalence of swine norovirus are very limited. The first detection of swine norovirus (Japan, 1997) occurred between February and July from faecal samples [19]. Two studies conducted in North America showed a higher prevalence in spring, particularly in March than in summer (June) or autumn (October) [43,57]. On the contrary, Silva et al. (2014) showed a higher prevalence during winter than in summer, although the difference was not significant [25]. Furthermore, few studies investigating the presence of antibodies against HuNoV and swine norovirus in swine are available. Farkas et al. (2005) conducted the first study on pig sera collected in the United States and Japan using virus-like particles (VLPs) based ELISA test [20]. Farkas et al., through VLPs, generated using a swine norovirus and a HuNoV (Norwalk prototype), showed a high prevalence of antibodies against both antigens in swine sera from the United States and Japan. Bucardo et al. investigated the seroprevalence in swine sera in Nicaragua between 2010 and 2015 [63]; antibodies against human genotypes GII.4 and GIII were detected in swine, and an increasing prevalence with the age of the tested animals was identified.

### 2.5. What: Genetic Diversity

**Figure 2:**
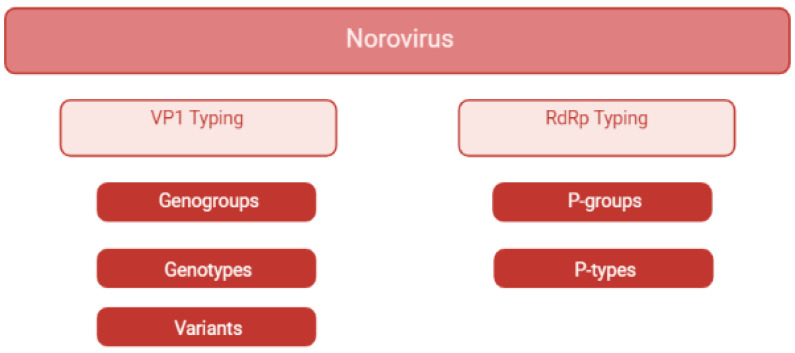
Classification scheme of noroviruses. Classification of noroviruses into genogroups, genotypes, variants, P-groups, and P-types, modified from Chhabra et al., 2019 [71]. Created in Biorender.com. (https://biorender.com/, accessed on 2 February 2022).

The current section describes the norovirus classification and the genotypes identified in swine. The first attempts to classify norovirus dates back to the nineties [71]. Initially, norovirus was classified into genogroups and genotypes according to the nucleotide sequences of the RdRp [71]. As more norovirus sequences became available, amino acidic VP1 sequences were considered more suitable for genotyping [71]. Currently, noroviruses are divided into 10 genogroups and 48 genotypes, and recently, two tentative genogroups (GNA1 and GNA2) and three new genotypes were proposed [71]. In detail, genotypes belonging to genogroups GI, GII, GIV, GVIII, and GIX are commonly detected in humans [13], while GII.11, GII.18, and GII.19 strain are specific to swine [13]. The GII.4 genotype, which causes human epidemics worldwide, is subdivided into different variants [72], as well as numerous strains belonging to all genotypes, frequently subject to recombination events. Norovirus has been detected in cattle and sheep (GIII), rats and mice (GV), dogs (GVI), and bats (GX). New tentative genogroups GNA1 and GNA2 were detected in harbour porpoise and sea lions, respectively [71]. In order to improve the classification of all these variants, in 2019, a new dual typing classification method was proposed [71], in which the ORF1- RdRp sequence defines the P-type, and the ORF2-VP1 sequence defines the genotype (Figure 2). The first swine norovirus detected in Japan in 1997 was recently classified as a GII.11 [19]. Strains belonging to genotypes GII.18 and GII.19 were described for the first time in the United States in 2005 [43]. After these first reports, the GII.11, GII.18, and GII.19 genotypes were described worldwide, and it is now commonly accepted that they are genotypes specific to swine. Notably, different genotypes not only proved to circulate in the same country, but high genetic diversity within the same genotype was also reported in strains circulating in the same geographical area and in the same period [28]. Despite this high genetic diversity, within each genotype, distinct genetic subgroups were identified and related to specific geographical regions [25]. In 2007, a human GII.4 strain was described in swine samples in Canada, raising concern about the role of swine as a reservoir for zoonotic norovirus [59].

Since then, the human GII.4 genotype was detected in swine in Japan in 2010 [35] and Taiwan in 2012 [46], together with other atypical genotypes, such as GII.3 and GII.13 in Japan and GII.23 and GII.21 in South Korea [69], while more recently the GII.2 genotype was detected in Dutch–German border region [55]. To date, the GII genotypes that commonly infect pigs (i.e., GII.11, GII.18, and GII.19) were not found in humans [70].

In conclusion, the most frequent genotypes identified in swine are GII.11, GII.18, and GII.19; however, others were detected, suggesting that further investigations are needed to assess the genotypes circulating in swine.

### 2.6. Phylogeny

**Figure 3:**
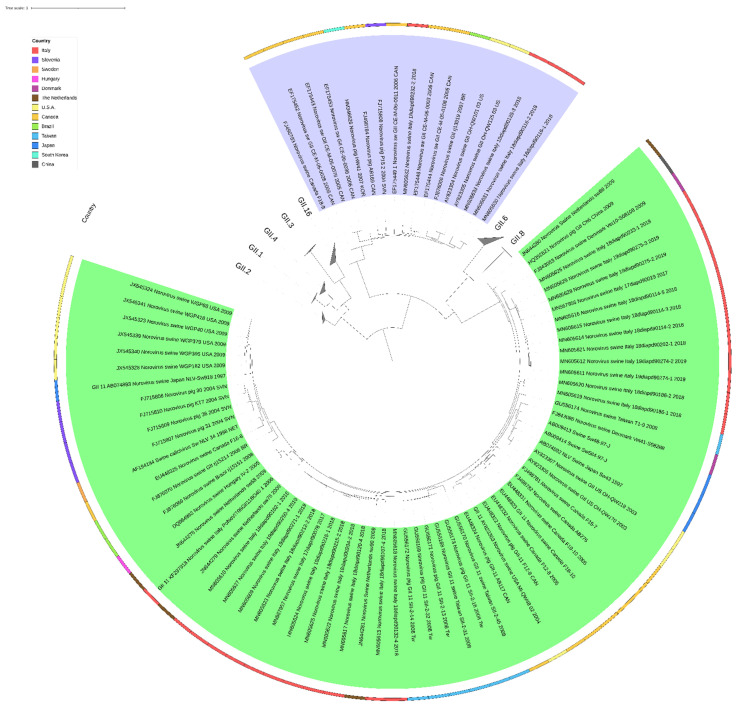
Phylogenetic tree, constructed with ML method and K2P model of nucleotide substitution, of a 300 bp RdRp gene fragment of the reference sequences of human and swine noroviruses available in GenBank, modified from Cavicchio et al., 2020. Swine strains (GII.11 and GII.18) are highlighted in green and purple, respectively.

Phylogenetic analyses are commonly carried out on a portion of the ORF1 and the ORF2, encoding for the RdRp and the VP1, respectively. Phylogenetic analysis might help to understand the dissemination of norovirus strains within a geographical region or between countries. However, few swine norovirus sequences are available due to a low number of studies on this virus and of the absence of standardised amplification protocols. This results in spatial and temporal gaps hampering evolutionary studies and any conclusions regarding the origin of swine noroviruses circulating in a specific area. Despite these limitations, the phylogeny based on the 300 bp portion of the ORF1 shows a clear separation between strains belonging to the GII.P11 p-type and those belonging to the GII.P18 p-type (Figure 3). Within the GII p-groups, the GII.P11 strains form a phylogenetically separated defined cluster from the other p-types; meanwhile, GII.P18 strains seem more related to HuNoV. This might represent a concern for human health; although noroviruses are generally considered to host specific species, the zoonotic potential of swine noroviruses has been and is still a subject of great interest. Within both the GII.P11 and GII.P18 p-types, strains of the same geographical origin tend to cluster together; however, distinct genetic subgroups can also be identified, suggesting the co-circulation of swine noroviruses with high genetic diversity in the same geographical area. Whether this high genetic diversity is the result of the independent introduction of swine noroviruses in a specific geographical area or represents the evolution from a common ancestor is still an open question. Furthermore, the circulation of different p-types and of genetically diverse swine noroviruses in the same area might lead to recombinant viruses with zoonotic potential. In this context, more efforts should be made to increase the number of available swine norovirus sequences, including those from archival samples, and to harmonise the molecular characterisation protocols. In the following figure is reported a phylogenetic tree constructed with the reference sequences of human and swine noroviruses based on a small fragment of the RdRp gene.

### 2.7. Swine Norovirus in Naturally Infected Swine

Studies on animal infectious diseases always face the question, “who is the target population?” For swine norovirus, some studies have investigated the correlation between the animal production category and/or age at sampling and the prevalence of swine norovirus [13,36,43,46,50,68]. Most of the studies that attempted to investigate this correlation were conducted retrospectively, and all swine categories were investigated, from piglets during weaning and post-weaning to finishers and sows. Such investigations require large sampling schemes to be statistically significant; however, due to the limited funds dedicated to animal noroviruses, it is difficult, if not impossible, to achieve the proper sample size. Several studies reported norovirus in both healthy and diseased pigs belonging to different productive categories; however, the observed clinical signs were nonspecific and could have been caused by other pathogens, hampering their correlation with norovirus infection.

### 2.8. Swine Norovirus in Experimentally Infected Swine

Only two studies described experimental infections using swine norovirus strains [43,50]. Wang et al. [43] inoculated two American GII.18 swine norovirus strains, namely QW126 and QW144, in gnotobiotic pigs. The faecal suspension of both swine noroviruses was inoculated (orally and intranasally) in one gnotobiotic pig each, of nine days of age. The two strains were replicated in gnotobiotic pigs, but while QW126 was detected in faeces only at five days post-inoculation (d.p.i.), the QW144 strain caused mild diarrhoea and a prolonged shedding (from 3 to 5 d.p.i.). In Shen et al., 2012 [50], swine norovirus faecal suspensions (1.5 mL) were used to infect five 15-day-old piglets through oral inoculation. Clinical signs (mild to moderate diarrhoea) started on 1 d.p.i. and persisted for two to six days, while no mortality was observed throughout the entire experiment. Interestingly, swine norovirus RNA was recovered from all faecal samples collected daily from 1 to 10 d.p.i., suggesting that piglets can shed the virus for a relatively long period. In conclusion, in vivo studies on swine norovirus would clarify several aspects of the infection; however, the unavailability of full sequenced viruses and the non-replicability on cell cultures represent bottlenecks for conducting such studies.

### 2.9. Host Factors

Pigs express HBGAs in different tissues, depending on the polymorphism of fucosyl transferase genes polymorphism. VLPs derived from HuNoV GI.P1 and GII.P4 genotypes were found to recognise A and H HBGAs in the duodenal and buccal tissues of pigs [73]. Furthermore, HuNoV belonging to genotypes GII.P4 and GII.P12 can replicate, cause disease, and induce an immune response in gnotobiotic pigs [14,74]. It was also observed that gnotobiotic pigs expressing A and H antigens shed significantly more HuNoV GII.P4 compared to pigs not expressing these antigens [73]. Lewis A (LeA) or Lewis B (LeB) HBGAs are associated with HuNoV susceptibility in humans, and LeB was found in gastric mucin and gastrointestinal tissue of pigs [18]. However, the frequency of these antigens in pig populations has not been investigated, nor has any putative association with HuNoV susceptibility in pigs.

### 2.10. Laboratory Methods to Investigate Swine Noroviruses

In the following sections, the laboratory methods commonly used for the detection, identification, and characterisation of swine norovirus are described, including the few information available on serological methods. Electron microscopy and RT-PCR/RT-qPCR were used for the detection of norovirus in faecal samples of animal origin. However, the high level of antigenic and genetic diversity among norovirus strains may influence the sensitivity and specificity of diagnostic tests [4]. Regarding serological methods, Enzyme-Linked ImmunoSorbent Assay (ELISA) tests based on VLP antigens were developed for the detection of swine norovirus antibodies in swine sera [20,43,68]. VLPs, expressing the norovirus VP1 protein of swine and human origin were generated and used to investigate the presence of norovirus antibodies in swine [20,43,68]. These assays have not been widely applied to investigate the circulation of swine norovirus in a target population because of their low sensitivity compared to nucleic acid detection methods and their low specificity due to the presence of antigenically distinct norovirus genotypes and the continuous antigenic drift of some strains. Therefore, data regarding the prevalence of swine norovirus positive serum in pigs are likely a misrepresentation of the real prevalence of swine norovirus infections in pigs.

### 2.11. Electron Microscopy

Electron microscopy (EM) was fundamental for the discovery of calicivirus in pigs [74]. However, EM is not a sensitive laboratory method [75]; the necessary instrumentation is expensive and requires well-trained personnel. Moreover, despite the characteristic morphology of all caliciviruses, norovirus is difficult to differentiate from other small enteric viruses commonly present in faeces [13]. Due to all the intrinsic limits of the method, ME is no longer used, and only the early studies on swine norovirus reported its use [19,21,22,43,47,58,60]. In Sugieda et al. [19,54], specimens were prepared for EM by cetyltrimethylammonium bromide as described in a previous study [53], using trihydroxy-dichloro-fluoroethane instead of trichloro-trifluoroethane. In Martinez et al. [47], samples were centrifuged through a 40% (*w*/*v*) sucrose cushion in PBS. The resuspended pellet was subjected to isopycnic centrifugation (110,000× *g*) in a CsCl gradient. Fractions with the expected density for caliciviruses (1.377 g/cm^3^) were desalted and concentrated by ultrafiltration. The samples were analysed under an electron microscope after negative staining with 1% uranyl acetate.

### 2.12. Pre-Treatment of Samples and RNA Extraction for PCR Detection

In the majority of studies, the presence of swine norovirus was investigated in faeces and intestinal contents since the latter represents the target tissue of the infection. Faeces and intestinal contents are complex matrices, with many inhibitors that may compromise the subsequent analytic steps; it is essential to identify the most effective pre-treatment and RNA extraction methods. Furthermore, norovirus is known to cause disease even with a low viral load; therefore, it is crucial to use an extraction method capable of isolating small quantities of viral RNA particles. A standardised pre-treatment method of faeces and intestinal contents for the isolation of the nucleic acids of swine norovirus is not yet available, and most studies rely on *in-house* protocols; however, the adoption of different methods of isolation and/or detection of the viral RNA hampers the identification of the most effective pre-treatment protocol. Usually, faeces are suspended in buffered saline solution (PBS) at 10% wt/vol, but the concentration could vary from 5 to 20% wt/vol [43,44,46]. Other studies reported the use of Minimal Essential Medium (MEM) [57,76], Hanks’ Balanced Salted Solution (HBSS) [56], 50% Freon [47], other viral transport mediums, of which the compositions were not specified, added with chloroform [60], Tris/HCl/Ca2+ [29,30] and ddh20 [27]. In order to complicate the scenario, the detailed composition and/or the pH values of the solutions used are rarely reported in the studies, hampering the correct reproduction of the methods. In most studies, samples were clarified through centrifugation, but they differ in respect to the speed (from 1700× *g* to 3000× *g*) and time of centrifugation, even though to high speed usually corresponds a short time and vice versa [29,30,31,32,34,46,56,59]. In some studies, the supernatant was then subjected to purification by a passage through microfilters (pore size of 0.22 μm) [53,64,65]. In a few studies, samples were only diluted in PBS without a centrifugation step [6,7,22,25,26,32,37,41], while only in one case, faecal samples were concentrated by ultracentrifugation (155,000× *g* for 2 h at +4 °C), and the pellet was suspended in sterile PBS [52]. In the early studies on swine norovirus, the RNA extraction was carried out using Trizol [31,32,34,38,39,43,47,53,67], which is not expensive, and allows to obtain a good yield of RNA from stools; however, it is time-consuming and potentially dangerous for the operator(s). More recently, commercial kits were preferred [31,33,41,49,50,65], and the Qiaamp Viral RNA mini kit (QIAGEN, Hilden, Germany) is the most commonly used [28,35,46,48,51,55,64]. Different internal control approaches are available for RNA viruses, such as (a) endogenous mRNA, (b) spiking of live viruses, or (c) synthetic RNAs in the samples prior to RNA isolation. Endogenous mRNAs were successfully used in the past, e.g., in assays for the detection of porcine reproductive and respiratory syndrome virus (PRRSV) [77]; however, the concentration of these mRNAs can vastly vary among clinical/field samples, hampering any quantitative analysis. Spiking of live viruses with a known titre seems an attractive option; however, it is a time-consuming approach, especially when dealing with a huge number of clinical/field samples, and there are concerns regarding safety and consistency [78]. Currently, synthetic RNAs may be the best option available as a safe, reliable, and robust IC; these RNA molecules of known sequence and concentration can be spiked in samples prior to RNA isolation, allowing controlling the entire process, both qualitatively and quantitatively [79].

### 2.13. RNA Transcription and Amplification Methods

Generally, the ORF1 encoding for RdRp is the target of molecular screening assays for swine norovirus detection. ORF1 is a conserved gene, and therefore it is well suited to this purpose. ORF2, encoding the VP1 protein, is used to characterise norovirus genotypes. Most studies describe a two-step approach, in which the reverse transcription phase is conducted separately from the cDNA amplification. In most studies the reverse transcription is conducted with random primers [29,30,32,36,40,41,46] or with specific primers. The latter are often used also for the amplification of cDNA [12,29,30,36,39,48,49,62,68]. In order to increase the performance of the molecular assays, in terms of sensitivity and inclusivity, more than one pair of primers were employed [19,29,30,31,34,41,54,57,60,62] and/or nested PCRs were frequently applied [24,32,65]. In some studies one-step RT-PCRs were adopted [22,24,28,36,44,48,55,61,64], while real-time RT-PCRs are rarely used [33,49,65,66]. Considering the advantages of real-time RT-PCR as a screening method in comparison to end-point PCR, or rather higher sensitivity and specificity, time-effectiveness, and reduced risk and contamination, more efforts should be invested in the development of effective and reliable real-time assays for the detection of swine norovirus. Furthermore, the adoption of a real-time multiplex approach for the simultaneous detection of a non-competitive internal extraction control (IC), or rather a target RNA amplified by a separate primer pair, might increase the robustness of the technique(s) used for the identification of swine norovirus, allowing distinguishing between false-negative results, due to inhibitors and/or human errors, and true negative results. Table 2 presents a list of primers commonly used for swine norovirus detection.

Primers p289/p290, originally developed for the detection of human norovirus and sapovirus, are widely used for the detection of swine norovirus; however, despite being highly inclusive, they showed a low specificity for some swine strains [13]. Indeed, this primer pair presents mismatches with their target sequences, hampering their detection [44,58]. Moreover, Ludert et al. demonstrated that these primers pair cross-reacts with rotaviruses [80]. Despite these issues, they are still considered the first choice for the detection of swine norovirus. A commonly accepted approach is to design and use several primer pairs based on the sequence of strains circulating in the area under investigation [13]. In the past, only the VP1 gene was used to characterise swine norovirus strains. In 2019, a new classification based on both VP1 and RdRp emerged but has not yet been formally applied to swine norovirus. In the literature, few primers for swine norovirus genotyping are described targeting the ORF2 [20,34,47,60]. However, the most used primer pairs for the characterisation of swine norovirus are G1SKF/G1SKR and G2SKF/G2SKR [35,36,41,46,50], specific for GII norovirus. Most authors preferred to fully sequence the VP1 gene to characterise swine norovirus strains.

### 2.14. Whole Genome Sequencing

Genetic studies in swine norovirus investigate either the genomic portion of ORF1, the gene that encodes for the VP1 protein (ORF2), and/or the ORF1/ORF2 junction, which is a known hotspot for recombination events between norovirus strains (see Figure 1). The common approach relies on RT-PCR followed by Sanger sequencing, using one of the protocols reported in Table 2 and discussed in the previous paragraph. However, other approaches can be adopted to obtain the entire genomic sequence of swine norovirus. In a recent publication, Laconi et al. [61] developed an *in-house* primer-walking strategy, obtaining the first European swine norovirus nearly full genome sequence and identifying an unusual potential recombination event in the ORF1. Clearly, the approach described by Laconi et al. is time-consuming, might not be effective against other swine norovirus strains, and relies largely on the quality and quantity of genetic materials. Okada et al. [36] obtained the nearly complete genome sequence of a swine norovirus strain detected in Japan in 2018 using next-generation sequencing (NGS). Indeed, in recent years, NGS has been successfully applied to virological investigations, including those on HuNoV; therefore, NGS seems to represent also the future of studies on swine noroviruses. However, NGS technologies are extremely sensitive to inhibitors, which are highly present in swine faeces, and are affected by the quality and quantity of the RNA in the samples. Therefore, a high concentration of inhibitors in combination with or without low RNA quality and quantity might hamper the good outcome of the NGS analysis. Some commercial kits for the enrichment of viral RNA are commercially available but have to be tested for norovirus. Moreover, in this respect, the identification of effective pre-treatment and isolation protocols of the nucleic acid is crucial. At the time of writing, only four complete genome sequences of swine norovirus are available in GenBank.

## 3. Conclusions

**Figure 4:**
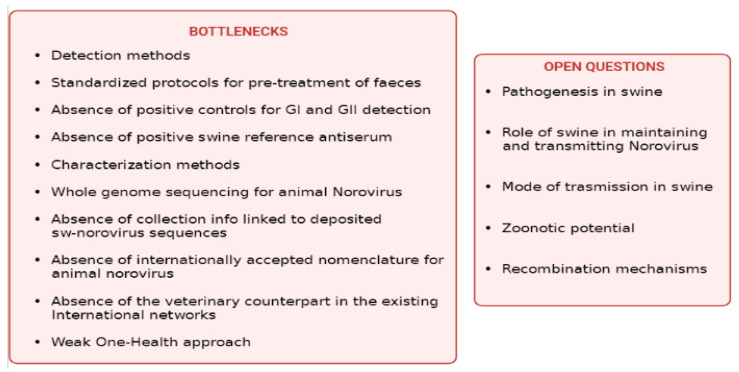
List of bottlenecks and open questions in studying swine Norovirus. Created in biorender.com. (https://biorender.com/), last accessed on 2 February 2022.

There are several bottlenecks in studying swine (and other animals) norovirus, including but not limited to technical issues in their detection and characterisation (Figure 4). In this respect, the unavailability of standardised methods and protocols for the pre-treatment of faeces, RNA isolation, and detection of positive controls against GI and GII representative P-types circulating in swine, and the absence of a reference swine serum for swine norovirus, further complicate the scenario. The deposit of swine norovirus genome sequences is of utmost importance to improve our knowledge of the epidemiology of swine norovirus and, in particular, to support the development of effective detection and characterisation methods. However, the publication of genomic data should be coupled with detailed data on collection and detection methods that are not often available. Regarding genome depositing, an additional technical bottleneck is how swine norovirus sequences are annotated, which may render the analysis and comparison of phylogenetic trees difficult. A common and internationally accepted nomenclature for animal norovirus should be adopted to facilitate the comprehensive inclusion of all available animal norovirus sequences and the interpretation of the phylogenetic trees. For swine norovirus sequences, Laconi et al. [61] and Cavicchio et al. [28] adopted a nomenclature resembling the one adopted for human and animal influenza viruses, including all the relevant strain information.

It is the authors’ opinion that the necessary steps to improve the methods available to study swine norovirus should be taken to increase the knowledge regarding this virus and to allow proper monitoring activities of its circulation and evolution in the swine population from a One-Health perspective. Networks on HuNoV and Caliciviruses exist at national and international levels but are not integrated with the veterinary world, hampering the flow of data between scientists and veterinarians. It is the authors’ opinion that this lack of exchange in knowledge between these two key figures might jeopardise the research on animal norovirus. Funds on veterinary virology are very limited, notwithstanding the importance it has in the study, prediction, and mitigation of zoonotic pathogens emerging and re-emerging diseases. Too frequently, national and international health organisations do not consider that the One-Health approach is applicable only if human and veterinary sectors are supported from financial and scientific points of view at similar levels.

Ultimately, from a research perspective, the availability of samples that are correctly stored, identified, and traced may lead to an intensive research activity, which may result in publishing and disseminating important epizoological data to the international scientific community. In this view, the collection and maintenance of good quality samples for swine norovirus research should be encouraged and supported, and scientific collections such as veterinary biobanks should receive more attention. This would allow addressing several existing open questions on swine norovirus such as the pathogenesis in swine, transmission mechanisms, the role of swine in maintaining and transmitting the virus, the zoonotic potential, the ability of swine norovirus to recombine. It is the authors’ opinion that the bottlenecks identified for swine norovirus also exist for other animal noroviruses and that the increase in knowledge in swine and animal “*norovirology*” would help understand the complex epidemiology of HuNoV. The future in studying swine norovirus looks like a blank wall unless more investments are available for non-notifiable animal infections and, in particular, for those with a putative zoonotic potential. Such a cultural change will remain a challenge, without coordinated actions among scientists in the area of veterinary virology, possibly with the foundation of national and international networks in the animal sector.

In conclusion, the approach and strategy that can be developed to study swine norovirus, taking into account bottlenecks and unanswered questions highlighted in the present review, may become a model for developing appropriate strategies to study several other animal infectious agents that share genetic similarities and ecological niches with human pathogens.

## Figures and Tables

**Table 1:** List of countries reporting swine norovirus in temporal order, sampling years, genotype, and reference.

Country	Sampling Years	Genotype	Paper
Japan	1997	GII.11	Sugieda et al., 1998 [19]
The Netherlands	1998	GII.11	Van der Poel et al., 2000 [56]
USA	2003	GII.18	Oka et al., 2013 [23]
Japan	2002–2003	GII	Farkas et al., 2005 [20]
USA	2003–2005	GII.18	Wang et al., 2006 [43]
Slovenia	2004–2005	GII.11, GII.18	Mijovski et al., 2010 [34]
Canada	2005–2007	GII.18, GII.4, GII.11	Mattison et al., 2007; L’Homme et al., 2009 [58,59]
Hungary	2005	NA	Reuter et al., 2007 [37]
Italy	2006–2007	GII.11	Di bartolo et al., 2014 [24]
New Zealand	2006–2007	GII	Wolf et al., 2009 [60]
Brazil	2007	GII.18	Cunha et al., 2010, Cunha et al., 2010 [29,30]
South Korea	2007–2009	GII.11, GII.18	Keum et al., 2009 [32]
Belgium	2007	GII.19	Mauroy et al., 2008 [44]
China	2008–2009	GII.19	Shen et al., 2012 [39]
Japan	2008–2009	GII.11, GII.18, GII.19, GII.3, GII.4, GII.13	Shen et al., 2009 [38]
Brazil	2008–2009	GII.11	Silva et al., 2015 [25]
USA	2009	GII.18, GII.11	Scheuer et al., 2013 [57]
Ethiopia	2013	GII.1	Sisay et al., 2016 [41]
Italy	2017–2018	GII.11, GII.18	Laconi et al., 2020, Cavicchio et al., 2020 [28,61]
Dutch–German border region	2017–2018	GII.2	Scheule et al., 2021 [55]
Japan	2017–2018	GII.11	Okada et al., 2020 [52]
Italy	2019	GII.11	Cavicchio et al., 2020 [28]

**Table 2:** List of oligonucleotide primers and probes for swine norovirus detection.

Primer Name	Sequence	Paper
For 35	5′-CTTGTTGGTTTGAGGCCATAT-3′	Sugieda et al., 1998 * [19]
Rev36	5′-ATAAAAGTTGGCATGAACA-3′	
For SMA82	5′-CCACTATGATGCAGATTA-3′	
REV NV81	5′-ATCTCATCATCACCATA-3′	
ReVNV82	5′-CACTATGATGCAGATTA-3′	
For JV12	5′-ATACCACTATGATGCAGATTA-3′	Van der poel et al., 2000 *, Mauroy et al., 2008nd [44,56]
Rev JV13	5′-TCATCATCACCATAGAAAGAG-3′	
For P290	5′-GATTACTCCAAGTGGGACTCCAC-3′	Wang et al., 2006 *†Δ, Martinezet al., 2006nd, Reuter et al., 2007 ◊, Martella et al., 2008nd, Mauroy et al., 2008nd, Schen et al., 2009 †, L’homme et al., 2009 *Δ, Halaihel et al., 2010nd, Nakamura et al., 2010 *†Δ, Chao et al., 2012 *, Shen et al., 2012 *, Scheur et al., 2013 *Δ, Sisay et al., 2013nd, Silva et al., 2014 *, Di Bartolo et al., 2014 *, Sisay et al., 2016 ◊, Valko et al., 2019 ◊, Stamelou et al., 2020nd [21,22,24,25,31,35,38,39,41,42,43,44,45,46,47,57,58]
Rev p289	5′-TGACAATGTAATCATCACCATA-3′	
For PNV7	5′-AGGTGGTGGCCGAGGAYCTCCT-3′	Wang et al., 2006 ◊, Scheur et al., 2013 *Δ, Sisay et al., 2013nd, Di Bartolo et al., 2014 *, Monini et al., 2015nd, Valko et al., 2019 ◊ [24,27,40,42,43,50]
Rev PNV8	5′-CACCATAGAAGGARAAGCA-3′	
Rev p289H,I-	5′-TGACGATTTCATCATCACCATA-3′ 5′-TGACGATTTCATCATCCCCGTA-3′	Wolf et al., 2009nd, Farkas et al., 2005 ◊, Martella et al., 2008nd, Cunha et al., 2010 *†Δ [20,21,22,29,30,60]
For p290H,I,J,K,	5′-GATTACTCCAGGTGGGACTCCAC-3′ 5′-GATTACTCCAGGTGGGACTCAAC-3′ 5′-GATTACTCCACCTGGGATTCAAC-3′ 5′-GATTACTCCACCTGGGATTCCAC-3′	
Monroe region B 431/433 For	5′-TGGACIAGRGGICCYAAYCA-3′ 5′-GAAYCTCATCCAYCTGAACAT-3′	Mattison et al., 2007 *Δ◊, Cunha et al., 2010 *†Δ, Gutierrez et al., 2011 ◊ [30,64,67]
Monroe region B 432/434 Rev	5′-TGGACICGYGGICCYAAYCA-3′ 5′-GGAYCGCATCCARCGGAACAT-3′	
Ando region A For G-2 SR46/G-1 SR48/G-1 SR50/G-1 SR52	5′-TGGAATTCCATCGCCCACTGG-3′ 5′-GTGAACAGCATAAATCACTGG-3′ 5′-GTGAACAGTATAAACCACTGG-3′ 5′-GTGAACAGTATAAACCATTGG-3′	Mattison et al., 2007 *Δ◊ [59] Mattison et al., 2007 *Δ◊ [59]
Ando region A rev G-1, G-2 SR33	5′-TGTCACGATCTCATCATCACC-3′	
Region C for MR3/Yuri22F	5′-CCGTCAGAGTGGGTATGAA-3′ 5′-ATGAATGAGGATGGACCCAT-3′	Wolf et al., 2009nd [60]
Region C rev MR4/Yuri22R	5′-AGTGGGTTTGAGGCCGTA-3′ 5′-CATCATCCCCGTAGAAAGAT-3′	
For swNo F	5′-AGGCAGCTCTATTGGACTAG-3′	Mauroy et al., 2008 † [44]
Rev swNo R	5′-GGTCTCATTATTGACCTCTGG-3′	
Rev P289N	5′-TCACGATTTCATCATCACCATA-3′	L’homme et al., 2009 *Δ [58]
For P290N	5′-GACTATTCACGGTGGGACTCCAC-3′	
For JV12Y	5′-ATACCACTATGATGCAGAYTA-3′	Mijovsky et al., 2010 *Δ, Halaihel et al., 2010nd [17,21]
Rev JV13I	5′-TCATCATCACCATAGAAIGAG-3′	
For P290	5′-GATTACTCCAAGTGGGACTCCAC-3′	Mijovsky et al., 2010 *Δ, Di Bartolo et al., 2014 *, Cavicchio et al., 2020 *Δ, Laconi et al., 2020 * [24,28,34,48]
Rev P110	5′-ACDATYTCATCATCACCATA-3′	
For COG2F	5′-CARGARBCIATGTTYAGRTGGATGAG-3′	L’homme et al., 2009nd [64]
Rev COG2R	5′-TCGACGCCATCTTCATTCACA-3′	
Probe RING2	5′-TGGGAGGGCGATCGCAATCT-3′	
For GIIF1 RT F RT	5′-GGGAGGGCGATCGCAATCT-3′	Keum et al., 2009 *◊ [32]
Rev GIIR RT, semi-nested R	5′-CCRCCIGCATRICCRTTRTACAT-3′	
For Cap GIIF2 Semi-nested F:	5′-TTGTGAATGAAGATGGCGTCGA-3′	
Song For	5′-GATTACTCCAGTGGACTTCCAAC-3′	Song et al., 2011 ◊ [69]
Song Rev	5′-TGACGATTTCATCATCACCCAGTA-3′	

Genotype detected with the couple of primers: * = GII.11, Δ = GII.18, † = GII.19, ◊ = other GII or calicivirus, nd = not detected.

## Data Availability

No new data were created or analyzed in this study.
